# Effect of DIO2 Gene Polymorphism on Thyroid Hormone Levels and Its Correlation with the Severity of Schizophrenia in a Pakistani Population

**DOI:** 10.3390/ijms25031915

**Published:** 2024-02-05

**Authors:** Farina Hanif, Quratulain Amir, Washdev Washdev

**Affiliations:** 1 Department of Biochemistry, Dow International Medical College, Dow University of Health Sciences, Karachi 74200, Pakistan; 2 Dow Institute of Medical Technology, Dow University of Health Sciences, Karachi 74200, Pakistan; quratulain.amir@duhs.edu.pk; 3 Dr. Abdul Qadeer Khan Institute of Behavioral Sciences, Dow University of Health Sciences, Karachi 74200, Pakistan; washdev.amar@duhs.edu.pk

**Keywords:** tetraiodothyronine (T4), triiodothyronine (T3), PANSS, genotyping, iodothyronine deiodinase 2, rs225014

## Abstract

Low levels of triiodothyronine (T3) in the brain lead to increased dopamine receptor sensitivity, potentially resulting in schizophrenia. Iodothyronine deiodinase 2 (DIO2) is the only enzyme which converts tetraiodothyronine (T4) to T3 in the brain. *DIO2* polymorphism of rs225014 results in the expression of non-functioning DIO2. Therefore, this study aimed to investigate the association of rs255014 with schizophrenia and its impact on thyroid hormone levels. This study included 150 schizophrenia cases and 150 controls. DNA was extracted from blood and subjected to PCR and amplicon sequencing. Serum thyroid profiles were determined using chemiluminescent magnetic microparticle immunoassay. Statistical analyses involved independent sample t-tests, Chi-square, and Pearson’s correlation tests. The results revealed a higher frequency of the reference genotype (TT) in controls compared to cases (*p* < 0.05). However, rs225014 did not influence serum thyroid levels or the severity of schizophrenia (*p* > 0.05). Interestingly, control subjects exhibited significantly higher T3 levels (*p* < 0.001) than cases. Regardless of the genotype (TT or CC), the control group had higher mean T3 levels than the corresponding case group (*p* < 0.05). In conclusion, rs225014 is associated with schizophrenia and has no effect on serum thyroid hormone levels.

## 1. Introduction

The brain needs an adequate supply of thyroid hormones to facilitate its metabolic and environmental adaptations [1]. An inherent homeostasis mechanism regulates local thyroid hormone levels [2]. Tetraiodothyronine (T4) is a physiologically inactive form of a thyroid hormone which enters the brain through the blood–brain barrier (BBB) and cerebrospinal fluid (CSF) barrier. Inactive T4 in the brain is locally converted to triiodothyronine (T3) through the action of the iodothyronine deiodinase 2 (DIO2) enzyme. Within the brain, thyroid hormones regulate the sensitivity of dopamine receptors and the activity of tyrosine hydroxylase, the rate-limiting enzyme of the catecholaminergic pathway, and myelination and inflammatory processes [3,4]. Apart from this, a hypothyroid state in the brain reduces cognitive abilities [5] and increases dopamine (DP) levels [6], an important etio-pathological factor of schizophrenia [7]. Decreased thyroid hormone levels have also been shown to increase dopamine receptor sensitivity and the severity of schizophrenia [8]. Additionally, dopamine agonists have been shown to decrease thyroid hormone levels [9].

Further, serum thyroid hormone levels are not representative of thyroid hormone levels in the brain [10]. Rodent studies have reported that serum T3 accounts for only 20% of T3 in the cerebral cortex; the rest is made available by the local conversion of T4 to T3 by the DIO2 enzyme, which regulates thyroid hormones under normal physiological and metabolic conditions. Studies have reported three different forms of DIO enzymes (DIO1, DIO2, and DIO3) with different physiological roles in the brain. DIO1 is not expressed in the brain. DIO3 has inhibitory effects: it converts T4 to rT3 and T3 into T2. Hence, in neural cells, DIO2 is the only enzyme available for the deiodination of T4 to T3 [10]. Therefore, normal *DIO2* expression is imperative to maintain normal T3 levels in the brain [9]. DIO2 is the primary and only available enzyme in the brain for T4-to-T3 conversion; its deregulation strongly impacts the brain’s levels of T3 but does not influence serum thyroid hormone levels because of the availability of DIO1 as well. It has been reported that serum thyroid hormone levels are not the true representative of brain thyroid hormone levels [3].

A single-nucleotide polymorphism (rs225014) in the *DIO2* gene has been found to be associated with impaired psychological well-being [11]. It is present at exon3 of the *DIO2* gene and results in the substitution of Thr92Ala. This substitution results in the ubiquitination of the enzyme DIO2, which is then no longer available to perform its normal physiological function, i.e., local conversion of T4 to T3. It has also been reported that *DIO2* rs225014 plays an important role in determining the brain’s ability to respond to serum T4 [11,12]. The importance of the *DIO2* gene in the regulation of brain thyroid hormone levels became more evident after studies reported that circulating thyroid hormone levels are not affected by rs225014 [11]. However, the *DIO2* gene does disrupt normal thyroid levels in the brain [9,13]. Hence, it can be assumed that the detection of *DIO2* polymorphism may be helpful in predicting the status of T3 levels in the brain. Consequently, previous studies have reported the association of *DIO2* rs225014 with bipolar disorder [14] and impaired psychological well-being [11]. However, to date, no study has examined the association of this polymorphism with schizophrenia and its effect on thyroid hormone levels in schizophrenia patients.

To the best of our knowledge, only one conference abstract is available showing the association of rs225014 (Trh92Ala) polymorphism with schizophrenia in a Turkish population [15]. No study has been conducted to date on *DIO2* gene polymorphism and its association with thyroid hormone levels in schizophrenia patients. Further, the abstract does not indicate the association of rs225014 variants with schizophrenia severity, nor does it analyze the impact of polymorphism on thyroid hormone levels. Therefore, the present study aimed to analyze the relationship of *DIO2* gene polymorphism with schizophrenia and its impact on the severity of the disease and thyroid hormone levels.

## 2. Results

### 2.1. Basic Demographics of Study Participants

Significant differences of age (*p* < 0.001) were found among the cases and controls (33.86 ± 9.63 and 29.5 ± 9.52). Mean age at disease manifestation was 25.67 ± 8.44 years, showing the onset of schizophrenia at a younger age. Regarding gender distribution among the case group, a significant difference was observed between male and female schizophrenia patients (*p* < 0.05). In the cohort of 150 schizophrenia patients, 72% were males and 28% were females (Table 1), indicating a difference in the prevalence of schizophrenia between the sexes. 

Various ethnic backgrounds were observed, and no significant differences were found between cases and controls (*p* = 0.344). Family history of a psychiatric disorder was found to be strongly associated with the onset of schizophrenia (*p* < 0.01). Further, a lower education status was found to be associated with schizophrenia (*p* < 0.001). Moreover, a higher education status was rarely found in the case group compared to the control group i.e., 26% vs. 74% respectively. A higher ratio of broken marriages was found in schizophrenia patients in this study, showing an association between divorce rate and schizophrenia (*p* < 0.001) (Table 2).

### 2.2. Genotyping

**Figure 1:**
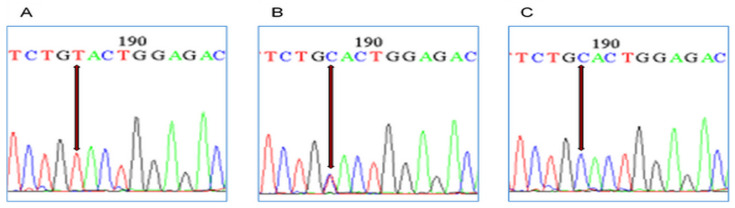
Electropherogram of representative regions of *DIO2* rs225014. (**A**) Arrows showing homozygous wild type (TT). (**B**) Arrow showing heterozygous (TC. (**C**) Arrow showing homozygous mutated (CC). The coloured peaks represent nucleotide bases of DNA. Blue, red, black and green represent Cytosine (C), Thymine (T), Guanine (G), and Adenine (A), respectively.

The electropherogram data generated after sequencing revealed all three variants of *DIO2*, rs225014, i.e., homozygous reference variant (TT), heterozygous (TC), and homozygous mutated genotype (CC). All three variants are shown in (Figure 1).

#### Genotype Frequencies of *DIO2* (rs225014) Gene Polymorphism in Schizophrenia Patients and Controls

The genotype distribution of rs225014 for the control group was in Hardy-Weinberg equilibrium (HWE) (*p*-value > 0.05). In contrast, the genotype distribution for the case group deviated from HWE (*p*-value < 0.05). Interestingly, a significant difference was found between *DIO2* (rs225014) gene polymorphisms and schizophrenia patients and controls (*p* = 0.019), with a higher frequency of the reference variant (TT) and heterozygous (TC) in controls and in cases, respectively (Table 3).

### 2.3. Association of PANSS Score with Genotype Frequency

To observe the effect of genetic polymorphism on the severity of psychotic symptoms in schizophrenia patients, the Positive and Negative Syndrome Scale (PANSS) was administered. Mean sub-scale scores for positive, negative, cognitive, and PANSS total scores were calculated and expressed along with standard deviations. A statistical analysis revealed no significant association of PANSS score with any of the genotype variants of *DIO2*, rs225014 (*p* > 0.05) (Table 4).

### 2.4. Thyroid Hormone Profile of Cases and Controls

Serum thyroid profiling (total T3, T4, and TSH levels) was done for both cases and controls. We found statistically significant differences in T3 levels between cases and controls (*p* < 0.001), with higher T3 levels in the control group. However, there was no significant difference regarding T4 and TSH levels among cases and controls (*p* = 0.817 and 0.691), respectively. A subgroup analysis with respect to gender between cases and controls revealed higher levels of T3 in males belonging to the control group (1.70 ± 0.24 vs. 1.50 ± 0.30) (*p* < 0.001). Moreover, significantly increased T3 levels were also found in females belonging to controls compared to those belonging to cases (1.53 ± 0.30 vs. 1.35 ± 0.37), respectively (*p* = 0.04) (Table 5).

#### Mean Levels of Thyroid Hormones with Respect to Gender

Significant differences (*p* = 0.004) were found with respect to T3 levels in both males and females, with higher T3 levels in males (1.6 ± 0.29) than in females (1.4 ± 0.34). Meanwhile, similar levels of T4 and TSH were observed in both genders. (Table 6).

### 2.5. Effect of DIO2, rs225014 on Thyroid Hormone Profile

A subgroup analysis was conducted to detect the effect of the individual variant of *DIO2*, rs225014, on serum thyroid hormone levels. Mean levels of T3, T4, and TSH were compared between subgroups based on the genotype. A statistically significant difference was found in mean T3 levels between cases and controls for individuals carrying the *DIO2*, rs225014 TT, and CC genotypes (*p* = 0.035 and 0.001), respectively, with higher mean T3 levels in controls with the TT and CC genotypes of the SNP. No significant difference in T3 levels was found in individuals with the TC genotype, rs225014, between cases and controls (*p* = 0.121). Regarding T4 levels, no significant difference was found between individuals carrying the TT, TC, and CC genotypes (*p* = 0.05, 0.464, and 0.417) between cases and controls. Further, *p* = 0.53 showed no significant difference among any of the genotype variants for *DIO2*, rs225014, or mean TSH levels both for cases and controls. Thyroid hormone levels were also compared among cases carrying different genotypes, and similar thyroid levels (*p* > 0.05) were found among individuals with different genotypes belonging to the case group (Table 7).

### 2.6. Correlation between PANSS Score and T3, T4, and TSH

To estimate the correlation between thyroid profile and severity of schizophrenia, we analyzed Pearson’s correlation between PANSS scores and the thyroid hormone profile (T3, T4, and TSH) of schizophrenia patients. No correlation was found among any of the PANSS subscale scores and serum levels of T3, T4, and TSH (Table 8).

## 3. Discussion

This is the first comprehensive study to report an association of *DIO2* rs225014 polymorphism with schizophrenia and its effect on serum thyroid hormone levels in schizophrenia patients. Our findings indicate a potential link between schizophrenia and variants of *DIO2* rs225014. We found a higher frequency of the reference genotype (TT) in the control group, inferring its protective effect against schizophrenia. Further, we also determined that *DIO2* rs225014 does not affect serum T3 levels.

Our analysis of demographic data revealed a higher prevalence of schizophrenia in males than in females among the recruited schizophrenia patients. This finding is consistent with other studies reporting a higher incidence of schizophrenia in males [16,17]. The presence of estrogen in females may be the underlying cause of this protective mechanism, ref. [18] as estrogen modulates the release and binding of dopamine to its receptors [19,20]. Moreover, estrogen therapy has also shown improvement in PANSS score in women suffering from schizophrenia [21]. As far as ethnicity is concerned, Urdu and Sindhi speaking individuals were recruited in larger numbers, probably because our single centered study was carried at IBS, DUHS, Karachi, Pakistan. Being a cosmopolitan city, Karachi’s inhabitants represent all ethnic groups in Pakistan, with the Urdu speaking population being the predominant group, followed by Punjabi, Sindhi, and other ethnic groups [22]. Individuals belonging to the Pathan, Balochi, Memon/Gujarati, Saraiki, and Hindko ethnicities were also recruited in the study, albeit in comparatively smaller numbers, and were therefore grouped into one group.

In relation to the effect of family history on psychiatric disorders, we found more of the participating patients with a positive family history of psychiatric illness compared to controls. This is also confirmed by previous twin studies and a number of other studies reporting a strong genetic association for schizophrenia [23,24].

Most patients in our schizophrenia case group were illiterate or had only received basic education, which may be due to their poor mental health and financial circumstances. This finding is consistent with the findings of Escott et al., who reported a lower level in academic achievement among individuals with schizophrenia [25]. Additionally, the control group in our study comprised peers and hospital staff, which might have contributed to the significant differences in educational level between the two groups.

Compromised cognitive abilities and the inability to meet the requirements of married life lead to increased marital disharmony, causing a higher divorce ratio in persons with mental illnesses such as schizophrenia [26,27,28]. The current study also found that schizophrenia patients had a greater divorce rate than the control group.

We also analyzed the effect of gender differences on thyroid hormone profile and found lower levels of T3 in females (*p* = 0.004). This observation is supported by studies reporting that a hypothyroid state is more common in females compared to males [29,30,31]. This observation contradicts the findings of other researchers who concluded that levels of T3 and TSH are not influenced by gender [32,33]. Further studies to reveal the impact of gender on thyroid hormone levels are therefore recommended.

Colak et al. also reported a higher frequency of the TT genotype in controls compared to the CC genotype in Turkish schizophrenia patients [15], indicating its protective effect against schizophrenia. However, their study was limited to an abstract, and it did not analyze the effect of *DIO2* rs225014 on the severity of the disease or on serum thyroid hormone levels. SNP rs225014 has been studied in other psychiatric disorders before, like bipolar disorder. For example, Bing He et al. reported a higher frequency of the T allele in the control group compared to the bipolar case group [14].

Further, it has been shown that a non-synonymous polymorphism of the *DIO2* gene (rs225014, T/C) replaces Thr92Ala at exon 3, resulting in the production of a physiologically inactive form of the DIO2 enzyme, subsequently leading to a hypothyroid state in the brain [11]. At this point, it should be kept in mind that DIO2 is the only enzyme available in the brain for the local conversion of T4 to T3 [10]. Further, this hypothyroid state in the brain affects the severity of psychological well-being [34,35]. Moreover, a strong relationship between *DIO2* rs225014 polymorphism (Thr92Ala) and bipolar mood disorder, mental retardation, hypertension, and the risk of osteoarthritis has also been reported by researchers [34]. Further, another study reported that hypothyroid patients with the CC genotype at rs225014 presented with better psychological wellbeing when given T3 therapy compared to T4 therapy. This further supports the rationale that patients with the CC genotype (92Ala) of rs225014 might have a hypothyroid environment in the brain, as T4 might not be converted to T3 because of the nonfunctional DIO2 enzyme. Many studies have shown altered levels of thyroid hormone in schizophrenia patients [36,37,38,39]. However, so far, no study has analyzed the association between *DIO2* gene polymorphism rs225014 and its effect on peripheral TH levels in schizophrenia patients.

Thyroid hormones (TH), both T3 and T4, are important for neural development, as well as for the day-to-day activities of the nervous system. DIO enzymes are required to convert T4 to T3. Low levels of thyroid hormone have been found to be associated with increased dopamine levels in schizophrenia patients; this is recognized as an important pathophysiological cause of schizophrenia [8]. Many treatment-resistant schizophrenia patients have shown improvement when given combined therapy of anti-psychotics and thyroid hormone [4,40]. Therefore, levels of thyroid hormones, especially in the brain, may help to determine the severity of the illness.

In the present study, we found similar levels of serum T4 and TSH between schizophrenia cases and controls. However, significantly low levels of serum T3 were found in cases compared to controls. Low levels of T3 in schizophrenia patients were also reported by Polat et al. [31], whereas the opposite result was reported by Singh et al. [41].

To find the effect of variants of *DIO2,* rs225014, on serum thyroid hormone levels, we conducted a subgroup analysis on serum thyroid hormone profiles (total T3, T4, and TSH) among cases and controls carrying different genotypes (TT, TC, and CC) of *DIO2,* rs225014. We found significantly low levels of mean T3 in both subgroups of cases carrying either the TT or the CC genotype compared to the respective control subgroups. These findings suggest that lower T3 levels in cases compared to controls are independent of this functional *DIO2* polymorphism, as subgroups carrying either the TT or the CC genotype had similar T3 levels, which may be because of the availability of other T4-converting enzymes in the periphery, like DIO1 [34,42]. Moreover, similar levels of T3, T4, and TSH were also reported in schizophrenia patients carrying the TT, TC, or CC genotypes (Table 7). Further, no significant difference was found in T3 levels between cases and controls with the TC genotype; this may have been because of heterozygosity, further confirming that serum T3 levels are independent of *DIO2* genotypes. Therefore, it is suggested that peripheral thyroid hormone levels, particularly T3, do not reflect the hypothyroid state of the brain, where only the DIO2 enzyme is available. Thus, detecting functional *DIO2* polymorphisms in schizophrenia patients is important. If patients with *DIO2* polymorphisms are given thyroid therapy, they should be given T3 rather than T4. Santos et al. also reported that the deregulation of DIO2 activity has no effect on circulating thyroid hormone levels but may affect the spatiotemporal distribution and regulation of thyroid hormone [3]. In our study, we found no significant difference regarding T4 and TSH levels in any of the subgroups of cases or controls.

To evaluate the relationship between the *DIO2* rs225014 polymorphism and the severity of schizophrenia, we administered PANSS to patients. A statistical analysis showed no significant association between variants of *DIO2* rs225014 (Thr92Ala) and the PANSS score. Replication studies with large sample sizes will be required to validate these findings. To the best of our knowledge, no previous study has discussed the association of this polymorphism with the PANSS score.

We also investigated the correlation of serum thyroid hormone levels (T3, T4, and TSH) with the severity of schizophrenia and did not find any correlation between the levels and PANSS subscale scores. This shows that serum thyroid hormone levels are not related to the severity of schizophrenia and supports our hypothesis that serum thyroid hormone levels do not reflect true schizophrenia pathology and symptoms. As such, the association of brain thyroid hormone with schizophrenia cannot be ruled out. However, it has been reported that the deregulation of DIO2 activity can influence the spatiotemporal distribution and regulation of TH, but it has no impact on circulating thyroid hormone levels [3]. These findings are in line with those of Telo et al., who did not find any correlation between PANSS subscale scores and levels of T3 and T4, although they reported a mild correlation between PANSS (negative symptoms score) and levels of TSH [43]. A few researchers have also reported a correlation between free T3 and improved cognitive function [44,45], suggesting that free T3 may improve cognitive function [44]. Again, these studies represented peripheral thyroid hormone levels, which are not a true representation of brain thyroid levels [9,11,13]. Our study concludes that *DIO2* polymorphism rs225014 is associated with schizophrenia but does not influence peripheral thyroid hormone levels.

Additional research focused on brain thyroid hormone levels is necessary to confirm the influence of *DIO2* polymorphism. This is crucial, because DIO2 is the sole enzyme in the brain responsible for converting T4 to T3, and approximately 80% of the intracellular T3 in the brain is generated locally through the deiodination of circulating T4 [11].

## 4. Materials and Methods

### 4.1. Ethical Approval and Consent to Participate

Our sample size was calculated using openepi.com with 80% power of test and a case-control study design. The calculated sample size was 135 individuals in each group, which was rounded up to 150 individuals per group [46]. The study was conducted according to the guidelines of the Declaration of Helsinki and approved by the Institutional Review Board, Dow University of Health Sciences Karachi, Pakistan. Informed written consent was obtained from patients or guardians or relatives of patients for participation in this study and the publication of clinical details.

### 4.2. Recruitment of Cases and Controls

A total of 300 participants, 150 cases and 150 controls, were recruited for this study from January 2018 to December 2021. Schizophrenia cases were recruited from the in-patient and outpatient departments of the Dr. Abdul Qadeer Khan Institute of Behavioural Sciences at the Dow University of Health Sciences. Diagnoses of patients with schizophrenia were carried out by a practicing psychiatrist following the guidelines of the Diagnostic and Statistical Manual of Mental Disorders-V (DSM-V) [47]. Patients with any other diagnosed endocrine or neurological disorders or pre-existing thyroid dysfunction were not included in the study. Controls without any diagnosed neurological, endocrinal, or psychological disorders were recruited from peers and hospital staff.

### 4.3. Demographic Details

Basic demographic details of study participants, like age, gender, ethnicity, education level, marital status, family history of any psychiatric disorder, medical history regarding any other comorbidity, duration of illness, and current medication, were recorded using a predesigned questionnaire.

### 4.4. Evaluation of Clinical and Psychopathological Symptoms

To assess the severity of illness, positive and negative syndrome scale scores (PANSS) were administered to patients by a psychiatrist, while the Beck Depression Inventory Scale II (BDI II) form was filled out by participating controls to rule out depression in the control group as one of the exclusion criteria.

### 4.5. Blood Collection and Thyroid Profile

Altogether, 6 mL of blood was drawn from each participant. Of this, 3 mL was collected in EDTA tubes and later used for DNA extraction. Another 3 mL was collected in gel tubes to obtain serum after centrifugation. The serum was separated and stored at −80 °C until used for thyroid profile assays.

Serum samples were sent to DOW Diagnostic Research and Reference Laboratories (DDRRL) where thyroid profile tests (total T3, T4, and TSH) were carried on an Abbott, ARCHITECT i2000SR via chemiluminescent magnetic microparticle immunoassay (Abbott Park, IL, USA).

### 4.6. DNA Extraction

DNA was extracted from whole blood using a DNA purification kit from Promega (Madison, WI, USA). as per the manufacturer’s protocol. The purity and quantity of DNA were checked using a nanodrop spectrophotometer (Thermofisher Scientific, Waltham, MA, USA). All DNA samples were then stored at −20 °C until further use for PCR.

### 4.7. Polymerase Chain Reaction (PCR) and Genetic Analysis

To amplify the genomic region encompassing *DIO2* (rs225014), the following primers were designed using the Primer 3 software: Forward (5′CCTCATCAATGTAGACCAGCAGGAA3′) and reverse (5′ AATGTGAATTCAAGTGGCAATGTGTTT3′). A reaction mixture with a final volume of 40 µL was prepared for each DNA sample or single PCR reaction. A negative control (containing all reagents except DNA) was also run to confirm that there was no contamination in the reaction mixture. Once the reaction mixture or master mix had been prepared, 38 µL of the mixture was transferred to each PCR tube (200 µL) which already contained 2 µL of DNA sample. The PCR tubes were then subjected to a short spin and transferred to a thermal cycler (Applied Biosystem SimpliAmp Thermal Cycler, Thermofisher Scientific, Waltham, MA, USA) for amplification. PCR was run according to the following conditions: initial denaturation at 95 °C for 5 min, followed by 35 cycles consisting of denaturation at 94 °C for 30 s, annealing at 54.5 °C for 45 s, extension at 72 °C for 45 s, and then final extension at 72 °C for 10 min.

### 4.8. Sanger Sequencing

After confirming specific amplification through gel electrophoresis, PCR products encompassing *DIO2* (rs225014) were sent to Macrogen Inc., Seoul, Republic of Korea, for purification and subsequent Sanger sequencing using ABI PRISM 3.0 Big Dye terminator chemistry.

### 4.9. Multiple Sequence Alignment of Nucleotides

The nucleotide sequences obtained for all samples were analyzed using MEGA 7.0 software. Sequences were matched with the reference sequence of *DIO2* (GenBank accession no: NC_000014.9) from the NCBI nucleotide data bank through the CLUSTAL W tool of the software [48], and allelic variants were identified.

### 4.10. Statistical Analysis

The data were analyzed using SPSS software version 21. Mean and standard deviation were used for continuous variables between cases and controls, while frequency and percentage were used for all categorical variables. To compare the means of the two groups, we performed an independent sample *t*-test. The Chi square test was used to compare the frequency (%) of two categorical variables. Pearson’s correlation was used to check the relationship between two continuous variables. A *p*-value < 0.005 was considered the level of significance.

## 5. Conclusions

The present study concludes that *DIO2*, rs225014, is associated with schizophrenia but does not affect serum thyroid hormone levels or the PANSS score. Further studies should be conducted to confirm the effect of *DIO2* polymorphism on CSF and brain thyroid hormone levels.

## Figures and Tables

**Table 1:** Age and gender distribution among schizophrenia cases and controls.

Characteristics	Cases	Controls	*p*-Value
	Mean ± SD	Mean ± SD	
Age (years)	33.86 ± 9.63	29.57 ± 9.52	<0.001
BMI	22.96 ± 5.75	23.20 ± 5.08	0.711
Age at Onset (year)	25.67 ± 8.44	-	-
Gender	N (%)	N (%)	0.028
Male	108 (72.0%)	90 (60.0%)	
Female	42 (28.0%)	60 (40.0%)	

*p* < 0.05 considered as statistically significant.

**Table 2:** Basic demographic features of study participants.

Characteristics	Cases N (%)	Controls N (%)	*p*-Value
Family history of psychiatric disorder			<0.001
Yes	47 (31.3%)	15 (10.0%)	
No	103 (68.7%)	135 (90.0%)	
Education			<0.001
Nil	44 (29.3%)	15 (10.0%)	
School	66 (44.0%)	24 (16.0%)	
College and above	40 (26.7%)	111 (74.0%)	
Ethnicity			0.344
Sindhi	47 (31.30%)	37 (24.7%)	
Punjabi	14 (9.3%)	21 (14.0%)	
Urdu Speaking	66 (44.0%)	73 (48.7%)	
Others	23 (15.3%)	19 (12.7%)	
Marital status			<0.001
Married	39 (26.0%)	52 (34.7%)	
unmarried	88 (58.7%)	97 (64.7%)	
Divorced	23 (15.3%)	1 (0.7%)	

*p* < 0.05 considered as statistically significant.

**Table 3:** Genotype frequency of *DIO2*, rs225014 among schizophrenia cases and healthy controls.

SNP ID	Genotype Frequency (%)			*p*-Value	*p*-Value (HWE)
rs225014	TT	TC	CC	0.019	
Cases	27 (18%)	91 (60.7%)	32 (21.3%)		0.008
Controls	44 (29.3%)	68 (45.3%)	38 (25.3%)		0.260

*p* < 0.05 considered as statistically significant. SNP: Single Nucleotide Polymorphism; Confidence Interval = 95%

**Table 4:** Association of PANSS score with genotype variants of *DIO2* rs225014.

PANSS Score	Positive		Negative		Cognitive		Total	
rs225014	Mean ± SD	*p*-Value	Mean ± SD	*p*-Value	Mean ± SD	*p*-Value	Mean ± SD	*p*-Value
		0.524		0.787		0.370		0.911
TT	63.5 ± 17.9		62.8 ± 16.7		73.9 ± 14.3		66.5 ± 15.7	
TC	68.9 ± 17.2		61.0 ± 15.5		67.8 ± 16.4		62.7 ± 17.0	
CC	66.8 ± 11.7		64.5 ± 19.1		71.0 ± 12.5		70.7 ± 14.5	

*p* < 0.05 considered as statistically significant.

**Table 5:** Mean levels of total thyroid hormones among males and females belonging to schizophrenia cases and controls.

Thyroid Hormone	Cases (Mean ± SD)	Controls (Mean ± SD)	*p*-Value
T3	1.46 ± 0.33	1.63 ± 0.28	<0.001
T4	7.52 + 1.69	7.46 ± 1.62	0.817
TSH	2.17 + 6.0	1.81 ± 6.02	0.691
Male			
T3	1.50 ± 0.30	1.70 ± 0.24	<0.001
T4	7.66 ± 1.62	7.29 ± 1.53	0.226
TSH	1.23 ± 0.66	2.10 ± 7.76	0.405
Female			
T3	1.35 ± 0.37	1.53 ± 0.30	0.040
T4	7.22 ± 1.83	7.72 ± 1.74	0.284
TSH	4.27 ± 10.6	1.36 ± 0.85	0.097

*p* < 0.05 considered as statistically significant.

**Table 6:** Mean levels of total thyroid hormones with respect to gender.

Gender	T3		T4		TSH	
	Mean ± SD	*p*-Value	Mean ± SD	*p*-Value	Mean ± SD	*p*-Value
		0.004		0.858		0.370
Male	1.6 ± 0.29		7.4 ± 1.58		1.6 ± 5.52	
Female	1.4 ± 0.34		7.5 ± 1.78		2.5 ± 6.78	

*p* < 0.05 considered as statistically significant.

**Table 7:** Difference in total thyroid hormone profile among genetic variants of *DIO2* (rs225014) between cases and controls.

Genotype	T3			T4			TSH		
	Cases Mean ± SD	Controls Mean ± SD	*p*-Value	Cases Mean ± SD	Controls Mean ± SD	*p*-Value	Cases Mean ± SD	Controls Mean ± SD	*p*-Value
TT	1.43 ± 0.33	1.61 ± 0.29	0.035	7.82 ± 1.61	6.91 ± 1.70	0.05	1.78 ± 2.13	1.35 ± 0.68	0.304
TC	1.51 ± 0.35	1.64 ± 0.31	0.121	7.33 ± 1.79	7.62 ± 1.40	0.464	1.69 ± 1.16	2.79 ± 9.75	0.531
CC	1.41 ± 0.30	1.65 ± 0.22	0.001	7.46 ± 1.65	7.84 ± 1.69	0.417	3.21 ± 10.83	1.05 ± 0.63	0.290
*p*-value	0.433	-	**-**	0.600	-	**-**	0.636		

*p* < 0.05 is considered as statistically significant.

**Table 8:** Pearson’s Correlation between PNASS score and thyroid hormone profile of schizophrenia cases.

Pearson Correlation	T3	T4	TSH
PANSS Positive	0.166	0.116	−0.060
PANSS Negative	0.040	0.179	0.032
General Psychopathology	−0.033	0.136	−0.013
PANSS Total	0.071	0.181	−0.015

## Data Availability

Data are contained within the article.
